# Supplementary material for: Flexible Ag2Se-based thin-film thermoelectrics for sustainable energy harvesting and cooling
Source: Nat Commun. 2025 Aug 15;16:7579. doi: 10.1038/s41467-025-62336-2 (PMC12354781; doi:10.1038/s41467-025-62336-2)
Supplement: Supplementary file 1 — Supplementary information [file 41467_2025_62336_MOESM1_ESM.pdf]

Supporting Information

Flexible Ag_2Se-based thin-film thermoelectrics for sustainable energy harvesting and cooling

Wenyi Chen [1,2], Meng Li [1], Xiaodong Wang [3], Joseph Otte [4], Min Zhang [1], Chengyang Zhang [1], Tianyi Cao [1], Boxuan Hu [1], Nanhai Li [1], Wei-Di Liu [1], Matthew Dargusch [2], Jin Zou [2,4], Qiang Sun [5,], Zhi-Gang Chen [1,*], Xiao-Lei Shi [1,*]*

[1] School of Chemistry and Physics, ARC Research Hub in Zero-emission Power Generation for Carbon Neutrality, and Centre for Materials Science, Queensland University of Technology, Brisbane, Queensland 4000, Australia.

[2] School of Mechanical and Mining Engineering, The University of Queensland, Brisbane, Queensland 4072, Australia.

[3] Central Analytical Research Facility, Institute for Future Environments, Queensland University of Technology, Brisbane, QLD, Australia.

[4] Centre for Microscopy and Microanalysis, The University of Queensland, Brisbane, Queensland 4072, Australia.

[5] State Key Laboratory of Oral Diseases & National Center for Stomatology & National Clinical Research Center for Oral Diseases, West China Hospital of Stomatology, Sichuan University, Chengdu, Sichuan 610041, P.R. China.

Corresponding Authors

*Xiao-Lei Shi: xiaolei.shi@qut.edu.au

*Qiang Sun: qiangsun@scu.du.cn

*Zhi-Gang Chen: zhigang.chen@qut.edu.au

1. Additional experimental details

Computational calculation: First-principles density-functional-theory (DFT) calculations were performed using a projector-augmented wave (PAW) method as implemented in the Vienna Ab initio Simulation Package (VASP) [1-5]. Fully relativistic Perdew-Burke-Ernzerhof generalized gradient approximation functional (GGA-PBE) was adopted to treat exchange correlation interaction [6]. A Monkhorst-Pack **k**-mesh of 80(250)/*a* (*a* is lattice constant) was created to sample the Brillouin space for structural relaxation (non-self-consistent calculations), with a plane wave cut-off energy of 500 eV. The convergence criterion for relaxation was set as 1×10^{-8} eV per electron and 1×10^{-3} eV·Å^{-1} per atom. The electron band structures were calculated along the line-mode **k**-path based on Brillouin path features indicated by the AFLOW framework [7]. The spin-orbital coupling effect was considered, with an initial magnetic momentum of 0.6 along the *z*-axis.

Single parabolic band (SPB) modelling: There are [8-11]:

$$S(\eta) = \frac{k_B}{e} \cdot \left[\frac{\left(r+\frac{5}{2}\right)\cdot F_{r+\frac{3}{2}}(\eta)}{\left(r+\frac{3}{2}\right)\cdot F_{r+\frac{1}{2}}(\eta)} - \eta \right] \tag{S1-4}$$

$$p = \frac{1}{e\cdot R_H} = \frac{(2m^*\cdot k_B T)^{\frac{3}{2}}}{3\pi^2\hbar^3} \cdot \frac{\left(r+\frac{3}{2}\right)^2 \cdot F^2_{r+\frac{1}{2}}(\eta)}{(2r+\frac{3}{2})\cdot F_{2r+\frac{1}{2}}(\eta)} \tag{S1-5}$$

$$\mu = \left[\frac{e\pi\hbar^4}{\sqrt{2}(k_B T)^{\frac{3}{2}}} \frac{C_l}{E_{def}{}^2 (m^*)^{\frac{5}{2}}} \right] \frac{(2r+\frac{3}{2})\cdot F_{2r+\frac{1}{2}}(\eta)}{\left(r+\frac{3}{2}\right)^2 \cdot F_{r+\frac{1}{2}}(\eta)} \tag{S1-6}$$

$$L = (\frac{k_B}{e})^2 \cdot \left\{ \frac{\left(r+\frac{7}{2}\right)\cdot F_{r+\frac{5}{2}}(\eta)}{\left(r+\frac{3}{2}\right)\cdot F_{r+\frac{1}{2}}(\eta)} - \left[\frac{\left(r+\frac{5}{2}\right)\cdot F_{r+\frac{3}{2}}(\eta)}{\left(r+\frac{3}{2}\right)\cdot F_{r+\frac{1}{2}}(\eta)} \right]^2 \right\} \tag{S1-7}$$

where η is the reduced Fermi level, k_B is the Boltzmann constant, r is the carrier scattering factor ($r = -1/2$ for acoustic phonon scattering), R_H is the Hall coefficient, C_l is the elastic constant for longitudinal

vibrations, and E_{def} is the deformation potential coefficient, respectively. There is [8-11]:

$$C_l = v_L^2 \cdot d \tag{S1-7}$$

The longitudinal velocity v_L is derived from the reference [12]. $F_i(\eta)$ is the Fermi integral, expressed as [8-11]:

$$F_i(\eta) = \int_0^{\infty} \frac{x^i}{1+e^{(x-\eta)}} dx \tag{S1-8}$$

The Maxwell–Eucken model was used for determining the intrinsic thermal conductivity of the Ag_2Se films [13]:

$$\kappa_{eff} = \frac{\kappa_m v_m}{v_m + \frac{3}{2} v_p} \tag{S1-9}$$

where κ_m is the thermal conductivity of the dense Ag_2Se matrix, and v_m and v_p represent the volume fractions of the matrix and pores, respectively.

2. Supporting figures

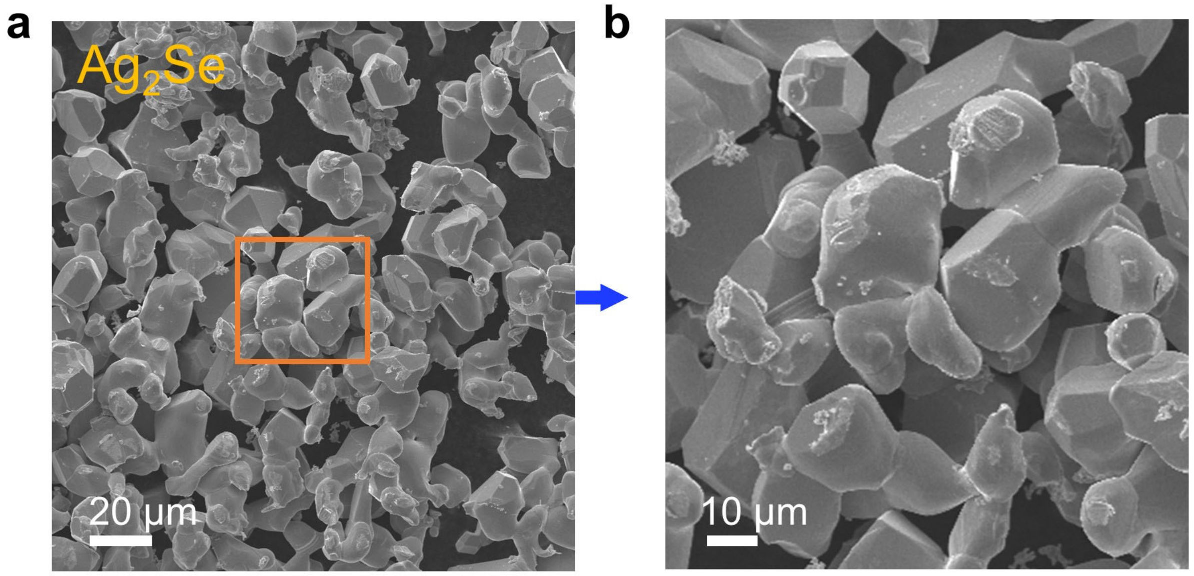

Supplementary Fig. 1 Scanning electron microscopy (SEM) images of Ag_2Se microparticles with **(a)** low- and **(b)** high-magnification.

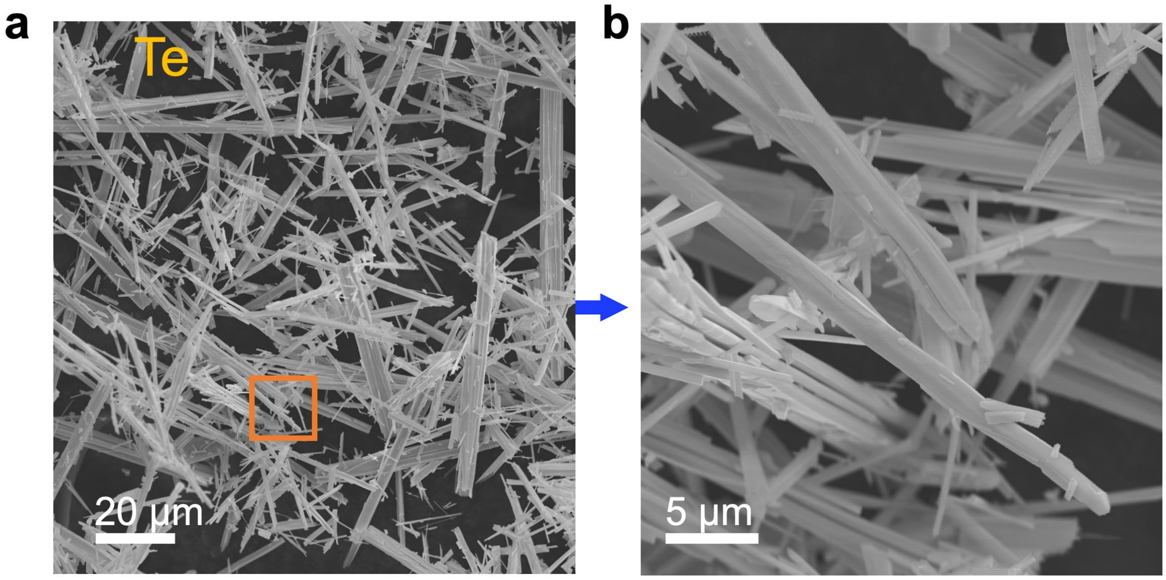

Supplementary Fig. 2 SEM images of Te nanorods with **(a)** low- and **(b)** high-magnification.

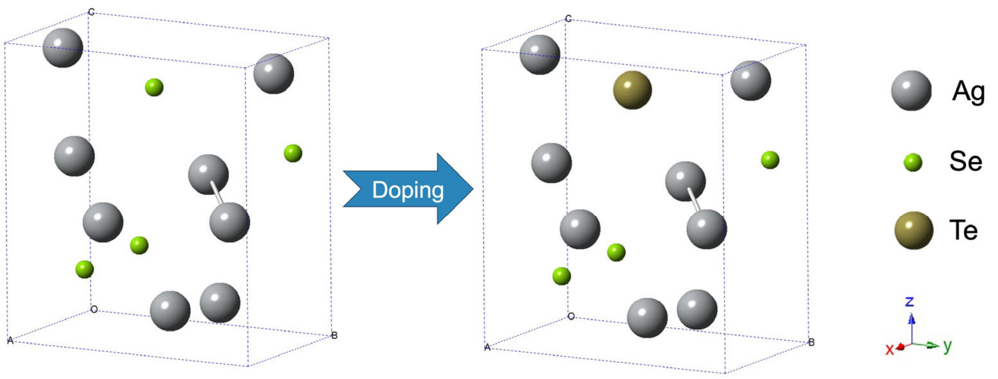

Supplementary Fig. 3 Schematic diagram of the unit-cell structure of the pure Ag_2Se and Te-doped Ag_2Se.

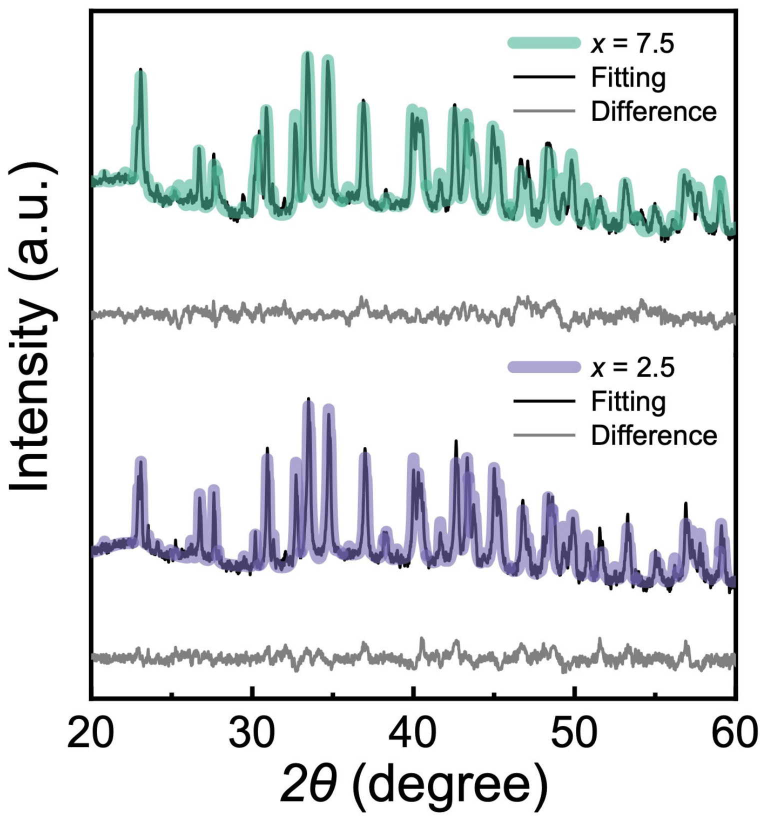

Supplementary Fig. 4 Rietveld quantitative phase analysis of Ag_2Se films with x = 2.5 and 7.5 wt.% Te.

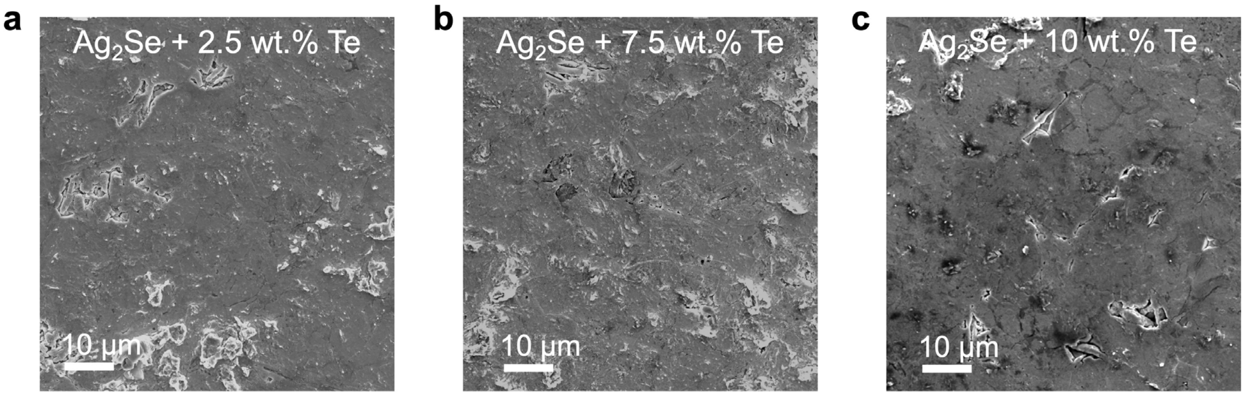

Supplementary Fig. 5 SEM images of Ag_2Se films with different Te contents x from top views: **(a)** x = 2.5 wt.%, **(b)** x = 7.5 wt.%, and **(c)** x = 10 wt.%.

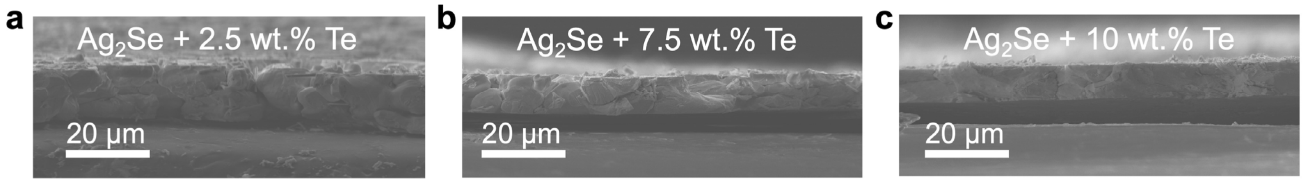

Supplementary Fig. 6 SEM images of Ag_2Se films with different Te contents *x* from cross-sectional views: **(a)** *x* = 2.5 wt.%, **(b)** *x* = 7.5 wt.%, and **(c)** *x* = 10 wt.%.

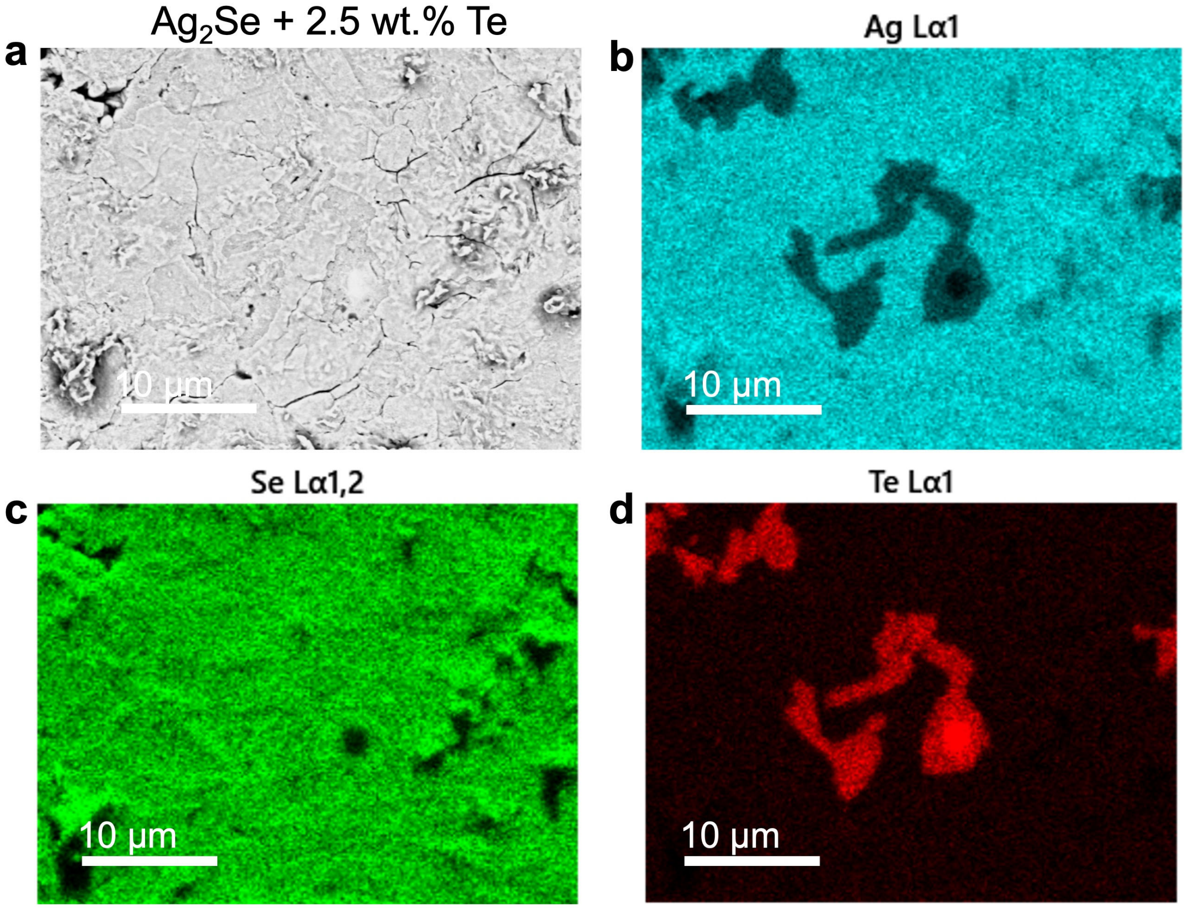

Supplementary Fig. 7 Characterizations of compositions of Ag_2Se films with 2.5 wt.% Te: **(a)** Backscattered electron (BSE) SEM images. Energy dispersive X-ray spectroscopy (EDS) maps for **(b)** Ag, **(c)** Se, and **(d)** Te.

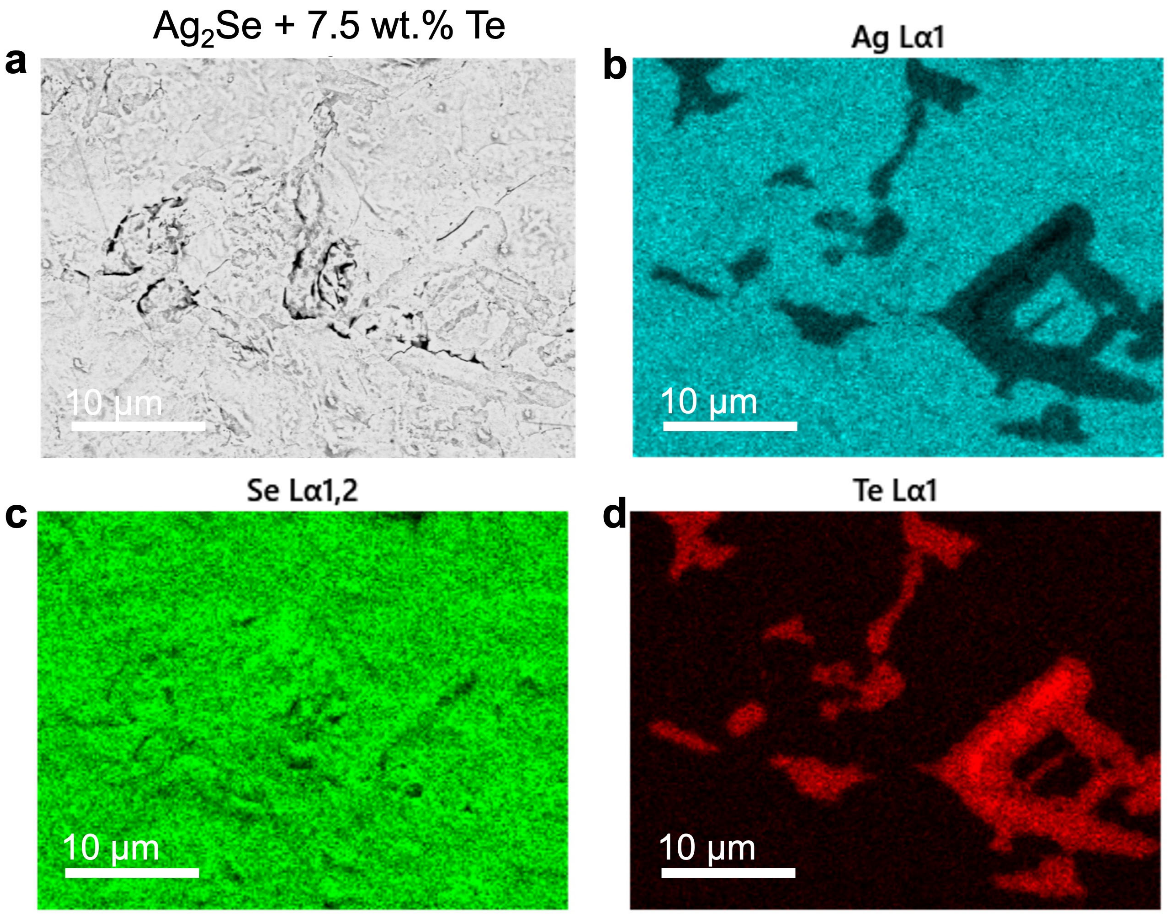

Supplementary Fig. 8 Characterizations of compositions of Ag_2Se films with 7.5 wt.% Te: **(a)** BSE SEM images. EDS maps for **(b)** Ag, **(c)** Se, and **(d)** Te.

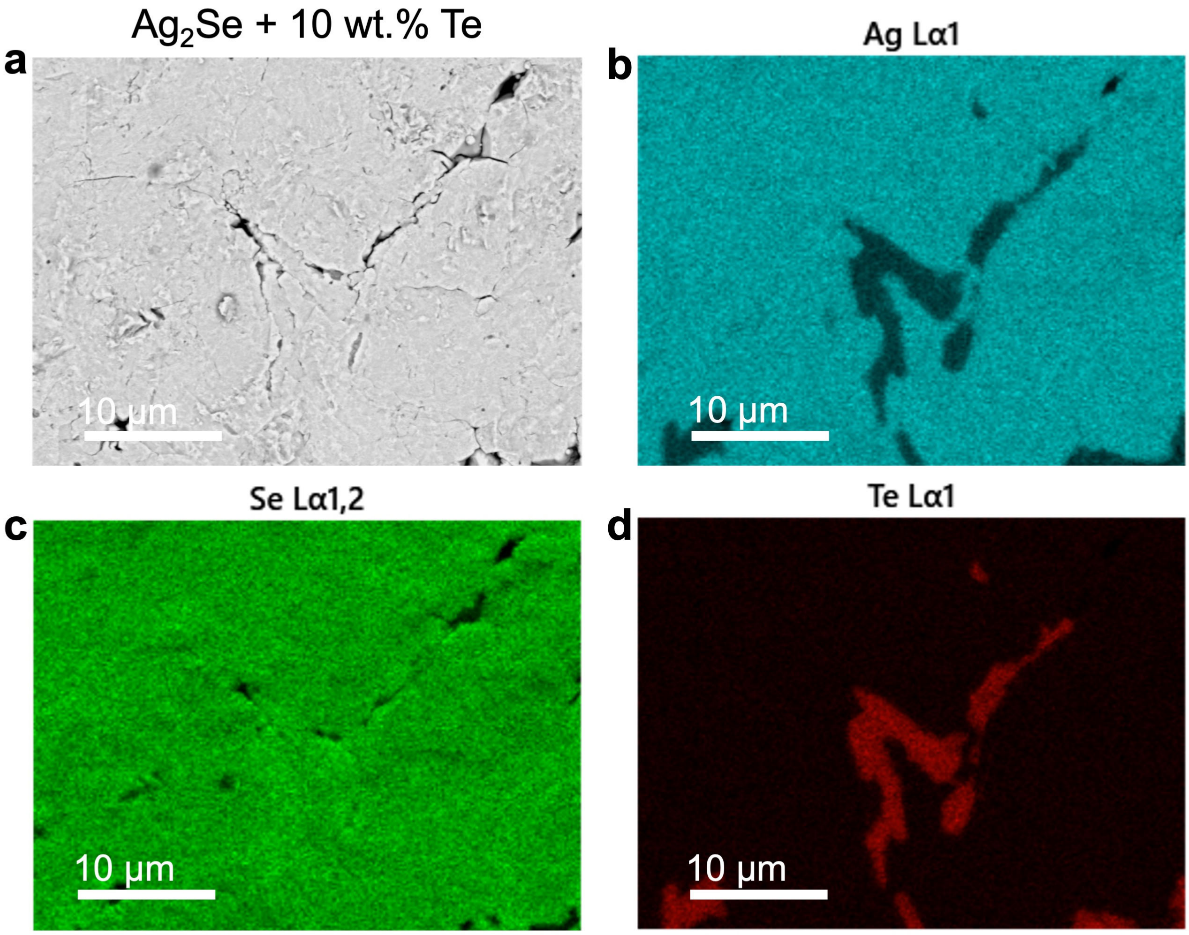

Supplementary Fig. 9 Characterizations of compositions of Ag_2Se films with 10 wt.% Te: **(a)** BSE SEM images. EDS maps for **(b)** Ag, **(c)** Se, and **(d)** Te.

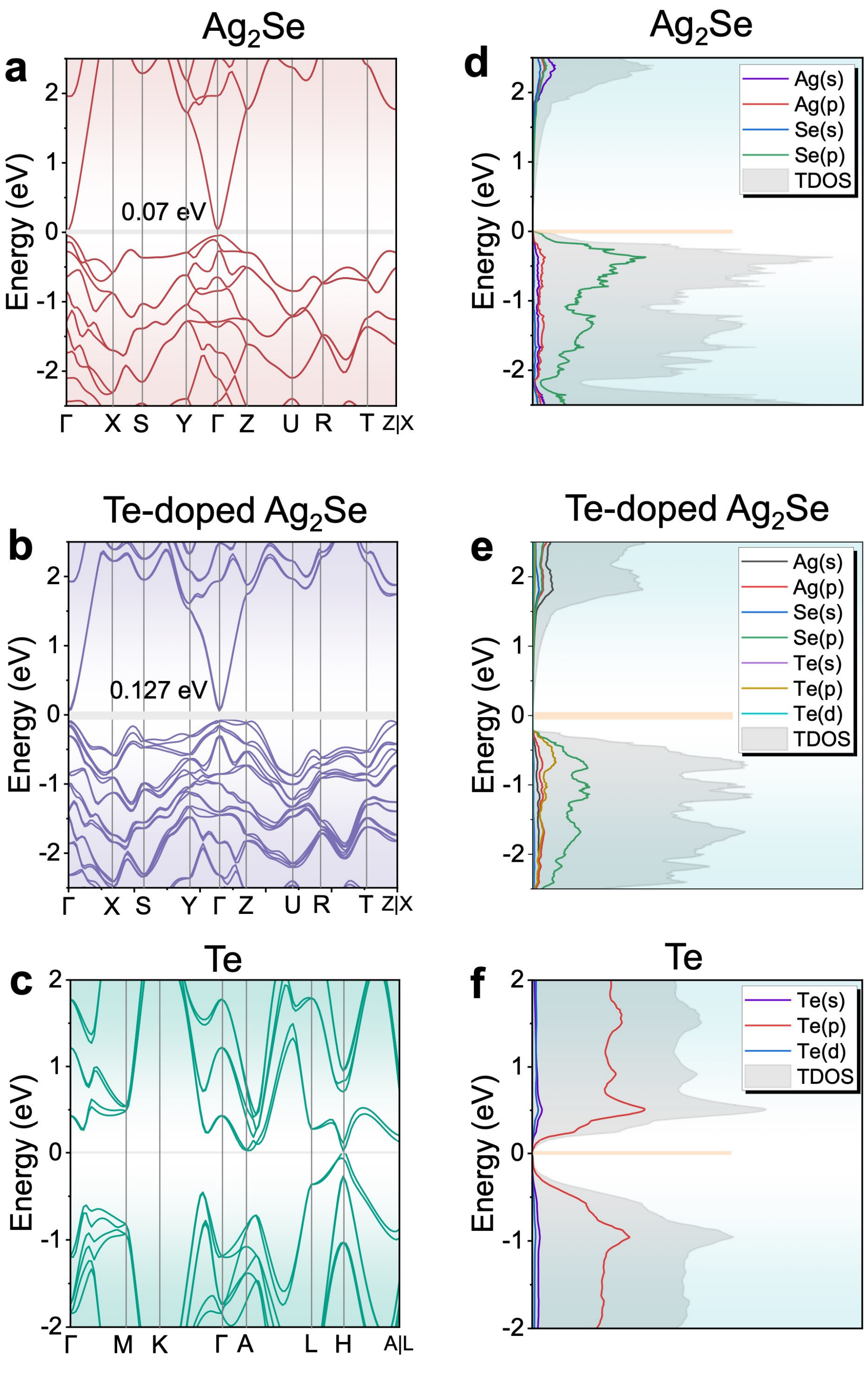

Supplementary Fig. 10 Calculated band structures and density of states (DOS) through density functional theory (DFT) calculations: Band structures of **(a)** Ag_2Se, **(b)** Te-doped Ag_2Se, and **(c)** Te. Corresponding DOS of **(d)** Ag_2Se, **(e)** Te-doped Ag_2Se and **(f)** Te.

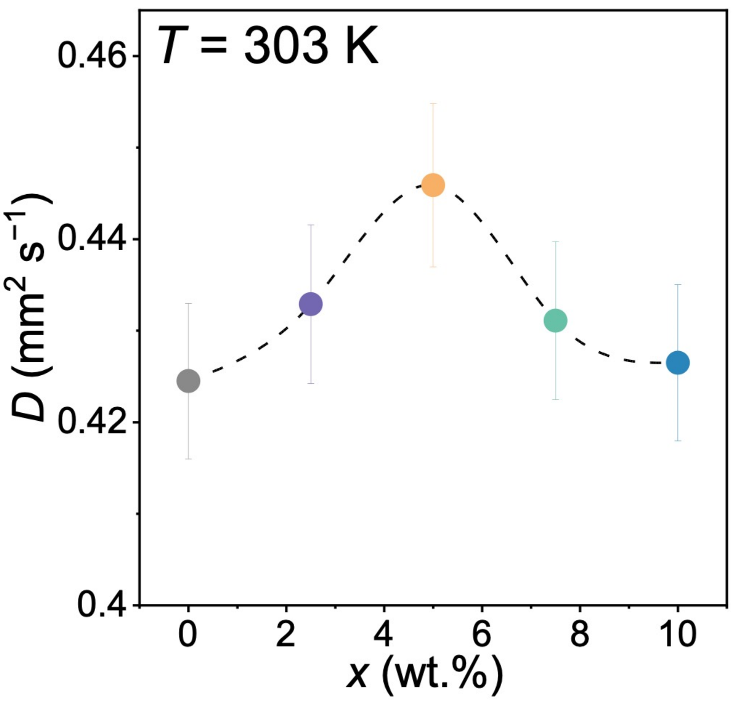

Supplementary Fig. 11 Thermal diffusivity (*D*) of Ag_2Se films with different Te contents (x = 0, 2.5, 5, 7.5, and 10 wt.%).

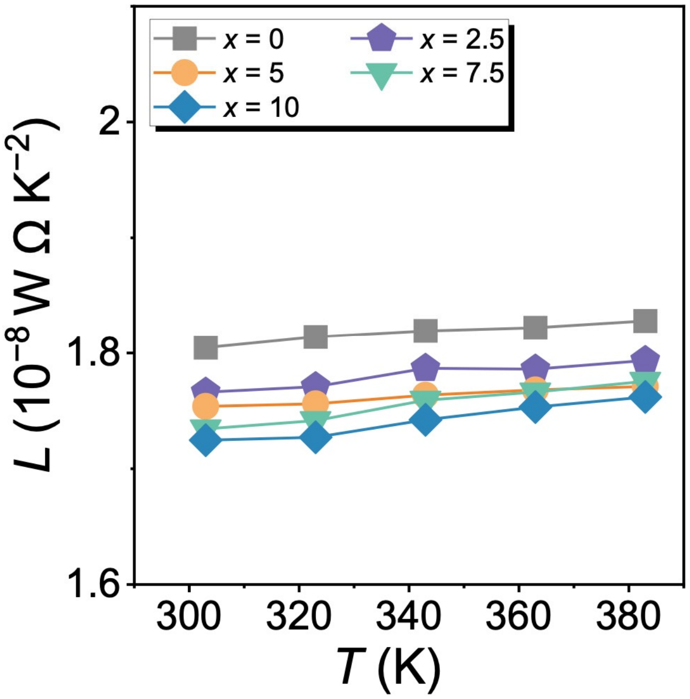

Supplementary Fig. 12 Temperature-dependent Lorentz number (*L*) of Ag_2Se films with different Te contents (x = 0, 2.5, 5, 7.5, and 10 wt.%).

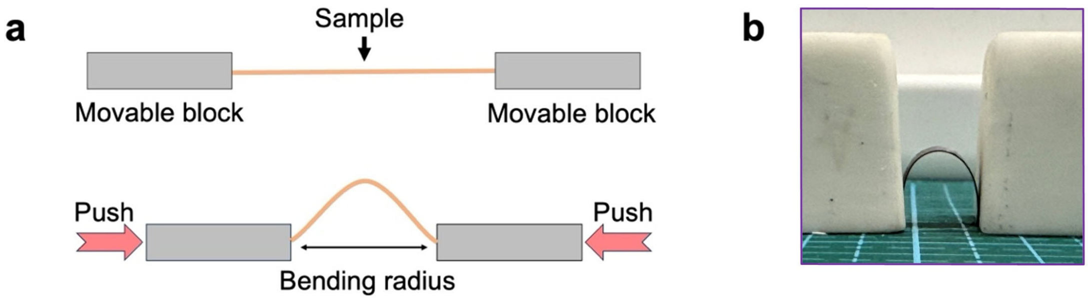

Supplementary Fig. 13 (**a)** Schematic of the bending test applied to the fabricated film. (**b)** Photograph of the experimental setup showing the film bent to a radius of 5 mm.

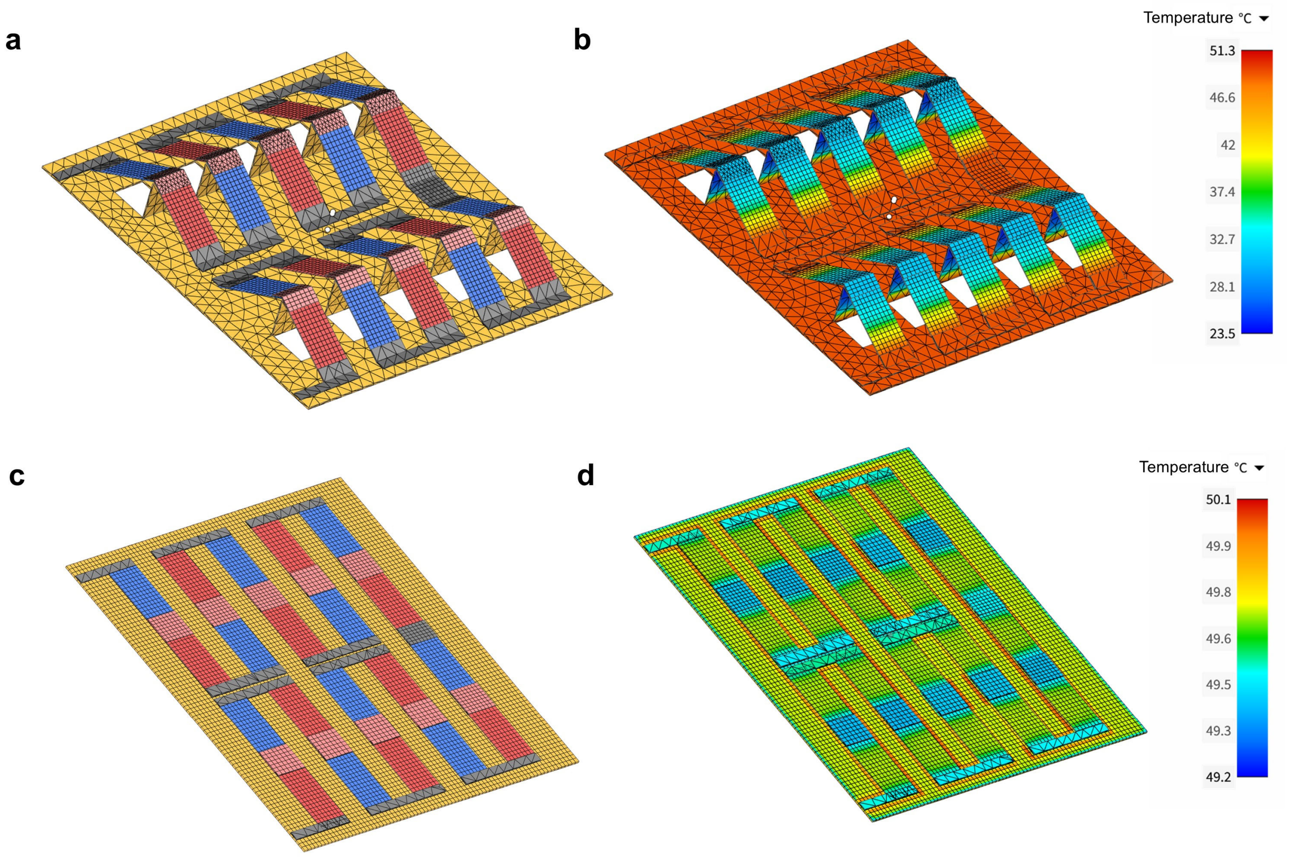

Supplementary Fig. 14 ANSYS simulations of thermoelectric devices. (a) Model of the as-fabricated device with a triangular structure, and **(b)** its simulated temperature difference. **(c)** Model of a device with a traditional parallel-leg structure, and **(d)** its corresponding temperature difference. Both device types use thermoelectric legs with identical dimensions. Simulations assume an ambient temperature of 22 °C and a heat convection coefficient of 5 W m^{-2} K^{-1}.

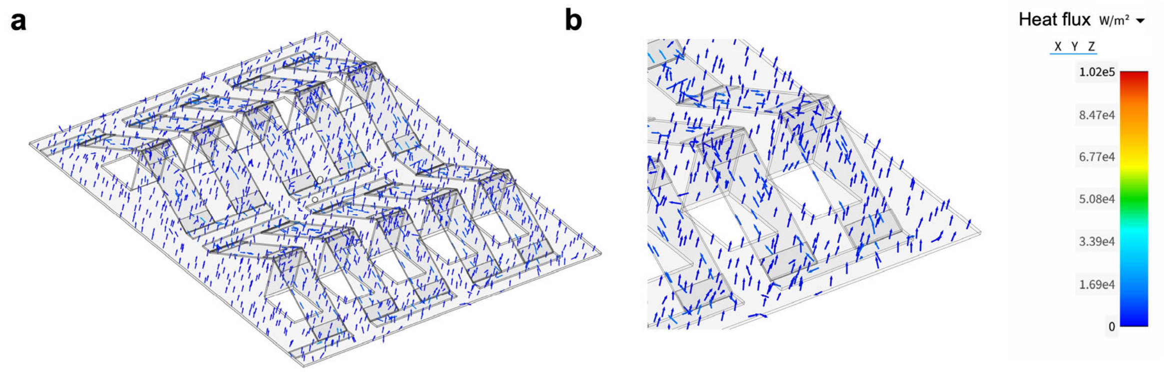

Supplementary Fig. 15 (a) Simulated heat flux distribution of the fabricated device. **(b)** Enlarged view of the heat flux flow direction from **(a)**. The ambient temperature is 22 °C, with a convective heat transfer coefficient of 5 W m^{-2} K^{-1}. The heat flux is uniformly distributed at approximately 8.45 × 10^{3} W m^{-2} across the device.

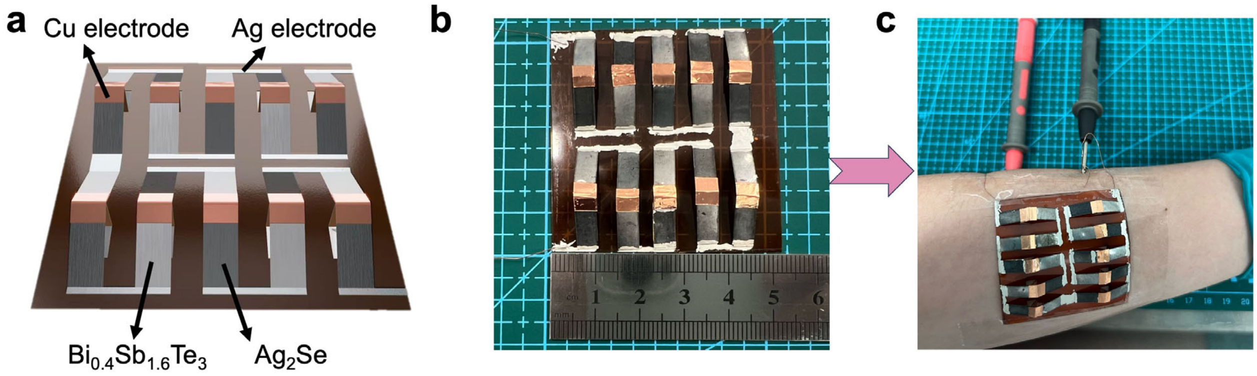

Supplementary Fig. 16 As-fabricated device: **(a)** schematic illustration of structure of as-fabricated device. **(b)** Photograph of as-fabricated device. **(c)** Photograph of as-fabricated device worn on human arm.

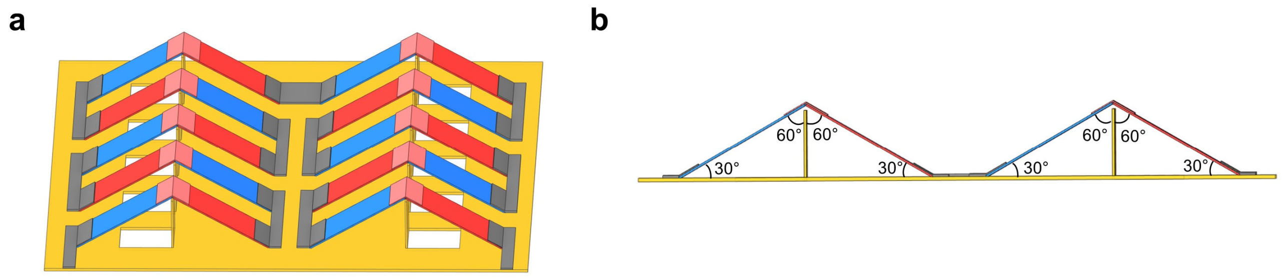

Supplementary Fig. 17 (a) Schematic of the modeled thermoelectric device. **(b)** Illustration of the angle at the triangular interface of the p–n junction. The model was developed using ANSYS.

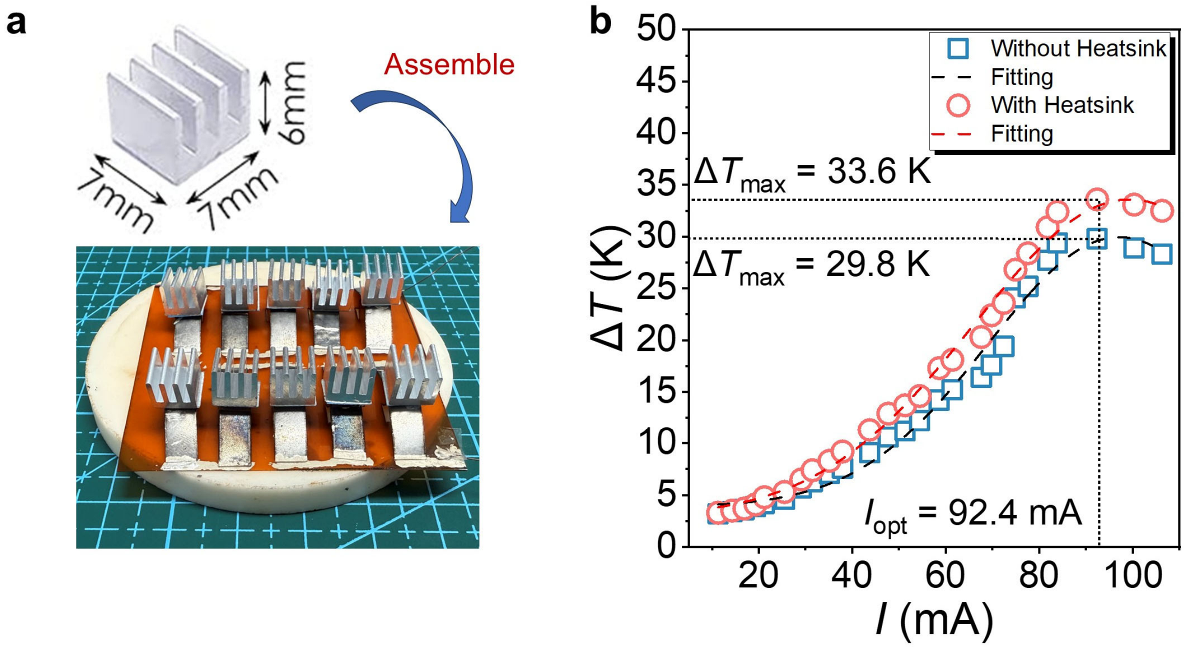

Supplementary Fig. 18 (a) Schematic illustration of the dimensions and structure of the commercial heat sinks and the as-fabricated F-TED with the heat sinks attached. **(b)** Comparison of the maximum temperature difference (ΔT_{max}) of the device with and without heat sinks as a function of input current.

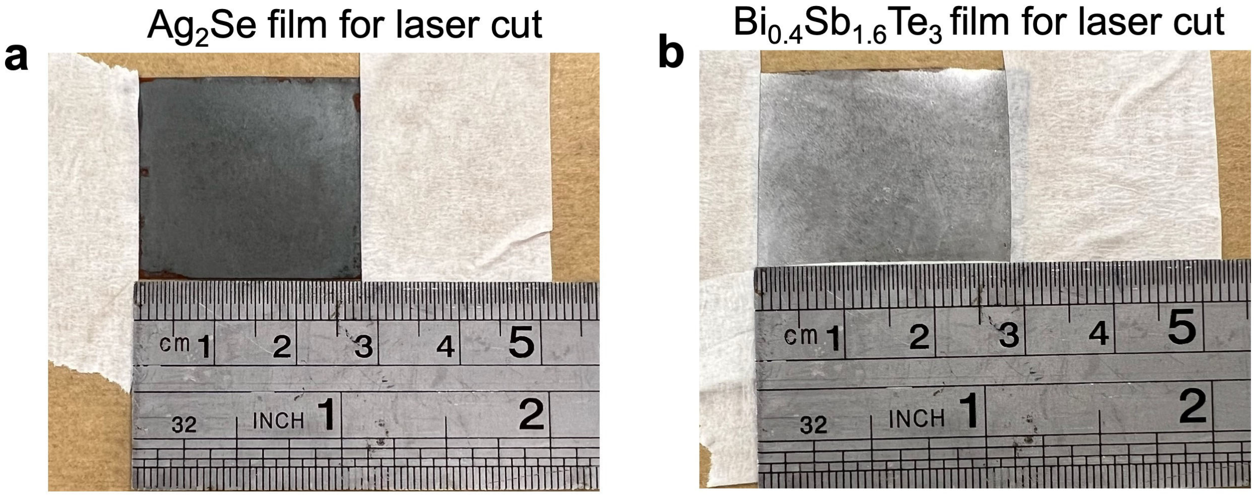

Supplementary Fig. 19 Photograph of the as-fabricated **(a)** n-type Ag_2Se film and **(b)** p-type $Bi_{0.4}Sb_{1.6}Te_3$ used for laser cut.

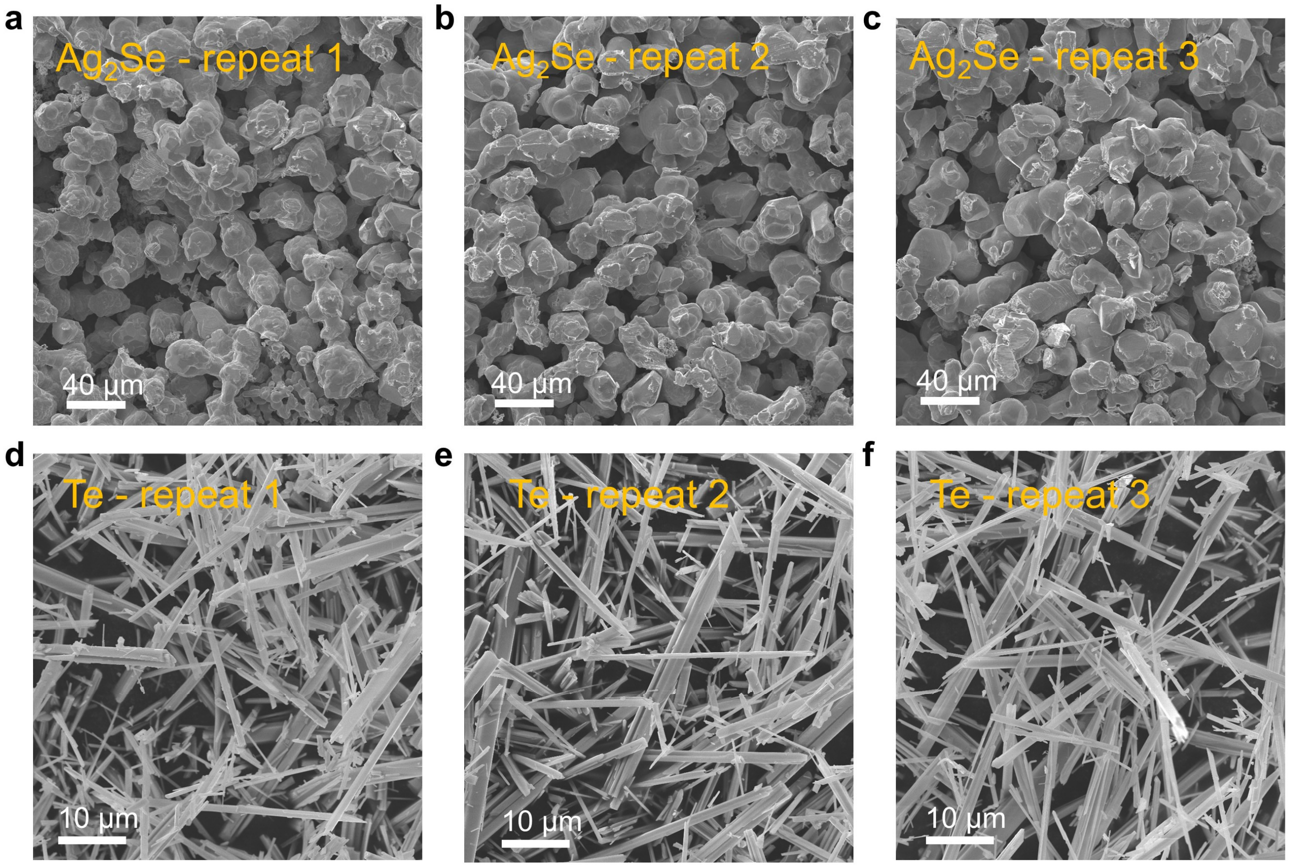

Supplementary Fig. 20 Scanning electron microscopy (SEM) images of **(a)**–**(c)** Ag_2Se microparticles and **(d)**–**(f)** Te nanorods synthesized in three independent trials, demonstrating consistent morphology and size across repetitions.

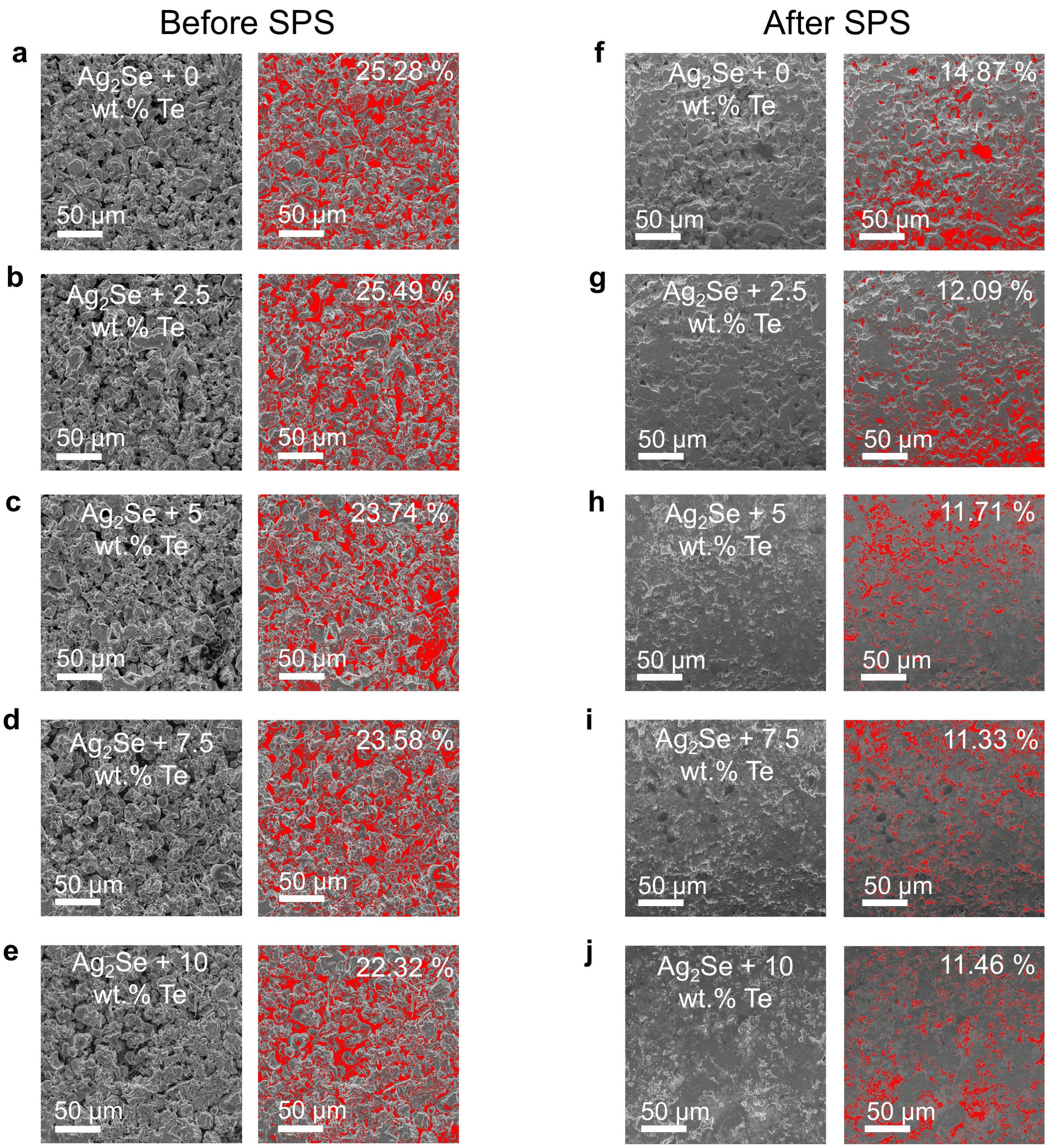

Supplementary Fig. 21 Scanning electron microscopy (SEM) images and corresponding porosity analysis of Ag_2Se films with 0, 2.5, 5, 7.5, and 10 wt.% Te content **a**–**e** before SPS and **f**–**j** after SPS. The porosity analysis is based on ImageJ.

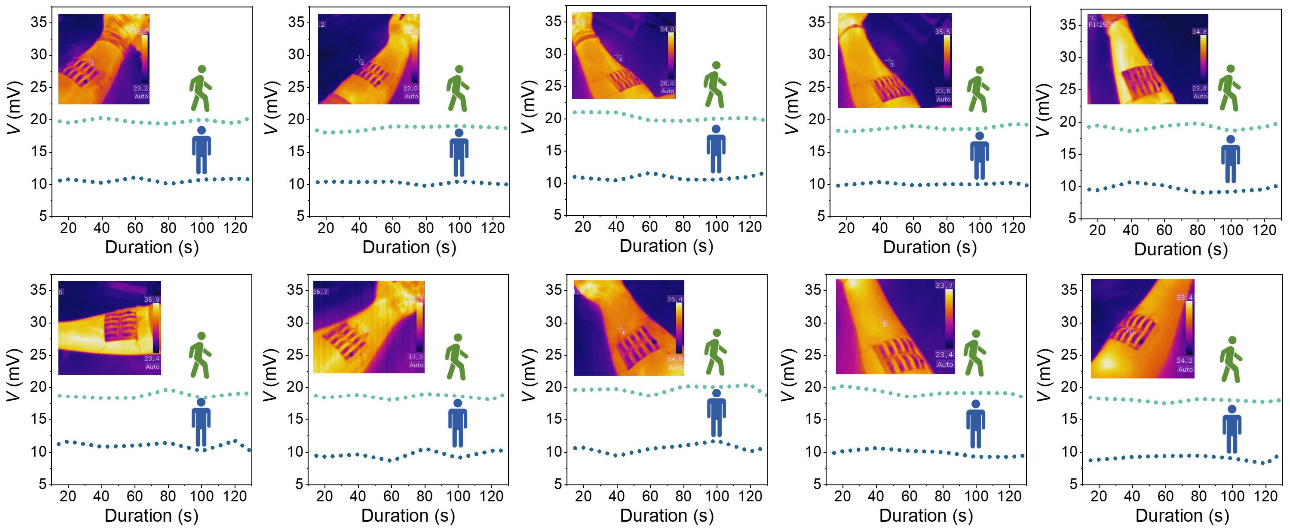

Supplementary Fig. 22 Experimental V_{oc} of the device worn on 10 different human arms during sitting and walking as a function of time (0~130 s). The inset displays an infrared photograph showing the temperature distribution of the device worn on the human arms while seated.

2. Supporting Table

Supplementary Table 1. Comparison of normalized power density (ω_n) between our screen-printed device and other reported flexible devices based on inorganic or hybrid thermoelectric materials. Here, poly(3,4-ethylenedioxythiophene) polystyrene sulfonate is abbreviated as PEDOT. Polyyvinyl alcohol is abbreviated as PVA. Single-walled carbon nanotubes are abbreviated as SWCNTs. Polypyrrole is abbreviated as PPy.

**Materials**		**Pairs**	**ΔT (K)**	**V_{oc} (mV)**	**P (μW)**	**ω (μW cm^{-2})**	**ω_n (μW cm^{-2} K^{-2})**	**Ref.**
**n**	**p**							
Ag-doped Bi_2Te_3	Sb_2Te_3	40	64	50	0.7	2100	0.51	14
$Ag_2S_{0.6}Se_{0.3}Te_{0.1}$	-	6	2.7	1.19	-	0.18	0.025	15
Ag_2Se	-	4	19.2	9.66	3.2	2200	5.96	16
Ag_2Se	-	125	40	30	0.8	-	2	17
Ag_2Se	-	4	50	28	0.48	4680	1.87	18
Ag_2Se	-	6	34.1	26.3	7.42	1.623	0.0014	19
Ag_2Se	-	40	-	15	-	1.7	-	20
Ag_2Se	-	5	25	12	-	-	-	21
Ag_2Se	Cu_2Se	36	40	60	-	1340	0.84	22
Ag_2Se	Sb_2Te_3	1	20	6	0.065	1500	3.75	23
Ag_2Se/Ag	-	8	27	18	7	874	1.19	24
Ag_2Se/Se	-	4	30	14.9	1.135	1.64	0.0018	25
Ag_2Se/Se/PPy	-	6	34.1	21.2	4.04	3.76	0.0032	26
Ag_2Te	-	16	30	8	0.101	-	0.9	27
Ag_2Te	-	4	50	18	-	-	-	28
Bi_2Se_3/SWCNTs	-	8	58	11.5	-	86.2	0.026	29
Bi-Te-Se	Bi-Sb-Te	3	5.7	-	-	58.3	1.79	30

Bi-Te-Se	Bi-Sb-Te	31	52	-	275	34.7	0.013	31
$Bi_2Te_{2.7}Se_{0.3}$	$Bi_{0.5}Sb_{1.5}Te_3$	18	50	236	4190	13100	5.24	32
$Bi_2Te_{2.7}Se_{0.3}$	$Bi_{0.5}Sb_{1.5}Te_3$	100	5.8	78.5	-	7.3	0.22	33
$Bi_2Te_{2.7}Se_{0.3}$	$Bi_{0.5}Sb_{1.5}Te_3$	32	11.7	33	-	29	0.21	34
$Bi_2Te_{2.7}Se_{0.3}$	$Bi_{0.5}Sb_{1.5}Te_3$	5	28.3	47.9	0.923	0.606	0.00076	35
$Bi_2Te_{2.7}Se_{0.3}$	$Bi_{0.5}Sb_{1.5}Te_3$	1	206	64	36	14.2	0.00033	36
$Bi_2Te_{2.7}Se_{0.3}$	$Bi_{0.5}Sb_{1.5}Te_3$	13	68	203	-	0.51	0.00011	37
$Bi_2Te_{2.7}Se_{0.3}$	$Bi_{0.5}Sb_{1.5}Te_3$	50	-	80	1200	48	-	38
$Bi_2Te_{2.7}Se_{0.3}$	$Bi_{0.5}Sb_{1.5}Te_3$	48	33.24	111.49	64.10	-	-	39
$Bi_2Te_{2.7}Se_{0.3}$	$Bi_{0.5}Sb_{1.5}Te_3$/Pb	5	10	8.5	0.026	-	-	40
Bi_2Te_3-based	Bi_2Te_3-based	440	-	-	-	-	0.26	41
Bi_2Te_3-based	Bi_2Te_3-based	-	-	40	-	12.1	-	42
Bi_2Te_3	-	6	50	11.4	0.069	0.0029	0.0000012	43
Bi_2Te_3	$Bi_{0.5}Sb_{1.5}Te_3$	256	2.73	191.6	-	0.090	0.012	44
Bi_2Te_3	$Bi_{0.5}Sb_{1.5}Te_3$	3	40	30	0.16	897.8	0.56	45
Bi_2Te_3	Co:ZnO	2	25	0.066	0.085	-	-	46
Bi_2Te_3	Sb_2Te_3	50	19	117	137.5	150	0.4	47
Bi_2Te_3	Sb_2Te_3	8	60	-	2	1420	0.39	48
Bi_2Te_3	PEDOT:PSS	32	45	-	9000	650	0.32	49
Bi_2Te_3/Carbon fibre	-	5	32	6.02	0.091	-	-	50
Bi_2Te_3/PVA	-	10	46	48	-	9	0.0042	51
Bi_2Te_3-SWCNT	-	5	25	17	0.055	930	1.49	52
-	BiSbTe	4	80	60	54	18800	2.93	53
-	Chitosan-Bi-Sb-Te	3	38	60	58	3500	2.42	54

-	Cu_2Se	-	500	38	-	621400	2.48	[55]
-	Cu_2Se	10	20	-	0.11	0.57	0.0014	[56]
Ni	Sb	10	50	37	0.12	4700	1.88	[57]
Ni-doped Bi_2Te_3	$Bi_{0.3}Sb_{1.7}Te_3$	220	52	1300	95900	610.8	0.23	[58]
Ni-doped Bi_2Te_3	$Bi_{0.3}Sb_{1.7}Te_3$	160	60	1000	77410	552.9	0.15	[59]
PEDOT/Ag_2Se/CuAgSe	-	11	36	46.8	3.21	840	0.64	[60]
PVDF/Bi_2Te_3	-	6	25	2.3	0.0126	-	-	[61]
PVP/Ag_2Se	-	6	29.1	28.72	4.16	28.8	0.034	[62]
$Mg_{3.15}Co_{0.05}SbBi_{0.99}Se_{0.01}$	$Yb_{0.9}Mg_{0.9}Zn_{1.198}Ag_{0.002}Sb_2$	2	430	150	505000	510000	2.76	[63]
Bi_2Te_3	Sb_2Te_3	282	40	91.73	6.65	370	0.23	[64]
Bi_2Te_3	Bi_2Te_3	76	47	525	406		1	[65]
Bi_2Te_3 commercial device	Bi_2Te_3 commercial device	-	62.5			63.83	0.016	[66]

Supplementary Table 2. Refinement results of GIXRD for Te-doped Ag_2Se.

Sample	Lattice parameter (*a*)	Lattice parameter (*b*)	Lattice parameter (*c*)
***x* = 0 wt.%**	4.3365	7.06936	7.7721
***x* = 2.5 wt.%**	4.3383	7.0705	7.7709
***x* = 5 wt.%**	4.3401	7.0763	7.7808
***x* = 7.5 wt.%**	4.3412	7.0808	7.7901
***x* = 10 wt.%**	4.3416	7.0822	7.7906

Supplementary Table 3. Thermoelectric properties of $Bi_{0.4}Sb_{1.6}Te_3$ films with 5 wt.% Te as p-type legs in thermoelectric device.

**Sample**	**Thickness**	***S*** **(μV K^{-1})**	***σ*** **(S cm^{-1})**	***S*^{2}*σ*** **(μW cm^{-1} K^{-2})**
$Bi_{0.4}Sb_{1.6}Te_3$ + 5 wt.% Te	≈ 20 μm	236.23	565.57	31.56

References

1. Kresse G, Hafner J. *Ab initio* molecular-dynamics simulation of the liquid-metal-amorphous-semiconductor transition in germanium. *Phys Rev B* **49**, 14251-14269 (1994).

2. Kresse G, Furthmüller J. Efficiency of *ab-initio* total energy calculations for metals and semiconductors using a plane-wave basis set. *Comp Mater Sci* **6**, 15-50 (1996).

3. Kresse G, Hafner J. Norm-conserving and ultrasoft pseudopotentials for first-row and transition elements. *J Phys-Condens Mat* **6**, 8245-8257 (1994).

4. Kresse G, Furthmüller J. Efficient iterative schemes for *ab initio* total-energy calculations using a plane-wave basis set. *Phys Rev B* **54**, 11169-11186 (1996).

5. Kresse G, Joubert D. From ultrasoft pseudopotentials to the projector augmented-wave method. *Phys Rev B* **59**, 1758-1775 (1999).

6. Perdew JP, Burke K, Ernzerhof M. Generalized Gradient Approximation Made Simple. *Phys Rev Lett* **77**, 3865-3868 (1996).

7. Setyawan W, Curtarolo S. High-throughput electronic band structure calculations: Challenges and tools. *Comp Mater Sci* **49**, 299-312 (2010).

8. Shi XL*, et al.* Boosting the Thermoelectric Performance of p-Type Heavily Cu-Doped Polycrystalline SnSe *via* Inducing Intensive Crystal Imperfections and Defect Phonon Scattering. *Chem Sci* **9**, 7376-7389 (2018).

9. Shi X*, et al.* High Thermoelectric Performance in p-type Polycrystalline Cd-doped SnSe Achieved by a Combination of Cation Vacancies and Localized Lattice Engineering. *Adv Energy Mater* **9**, 1803242 (2019).

10. Shi X*, et al.* Polycrystalline SnSe with Extraordinary Thermoelectric Property *via* Nanoporous

Design. *ACS Nano* **12**, 11417-11425 (2018).

11. Jin M*, et al.* Super Large $Sn_{1-x}Se$ Single Crystals with Excellent Thermoelectric Performance. *ACS Appl Mater Interfaces* **11**, 8051-8059 (2019).

12. Liu M, Zhang X, Zhang S, Pei Y. Ag_2Se as a tougher alternative to n-type Bi_2Te_3 thermoelectrics. *Nat Commun* **15**, 6580 (2024).

13. Wang J, Carson JK, North MF, Cleland DJ. A new approach to modelling the effective thermal conductivity of heterogeneous materials. *Int J Heat Mass Tran* **49**, 3075-3083 (2006).

14. Zheng Z-H*, et al.* Harvesting waste heat with flexible Bi_2Te_3 thermoelectric thin film. *Nat Sustain* **6**, 180-191 (2023).

15. Liang J, Zhang X, Wan C. From Brittle to Ductile: A Scalable and Tailorable All-Inorganic Semiconductor Foil through a Rolling Process toward Flexible Thermoelectric Modules. *ACS Appl Mater Interfaces* **14**, 52017-52024 (2022).

16. Jiang C*, et al.* Ultrahigh Performance of n-Type Ag_2Se Films for Flexible Thermoelectric Power Generators. *ACS Appl Mater Interfaces* **12**, 9646-9655 (2020).

17. Liu Y*, et al.* Fully inkjet-printed Ag_2Se flexible thermoelectric devices for sustainable power generation. *Nat Commun* **15**, 2141 (2024).

18. Hou S*, et al.* High performance wearable thermoelectric generators using Ag_2Se films with large carrier mobility. *Nano Energy* **87**, 106223 (2021).

19. Zhang M*, et al.* Scalable printing high-performance and self-healable Ag_2Se/terpineol nanocomposite film for flexible thermoelectric device. *Energy* **296**, 131232 (2024).

20. Liu Y*, et al.* Scalable-produced 3D elastic thermoelectric network for body heat harvesting. *Nat Commun* **14**, 3058 (2023).

21. Palaporn D, Mongkolthanaruk W, Faungnawakij K, Kurosaki K, Pinitsoontorn S. Flexible Thermoelectric Paper and Its Thermoelectric Generator from Bacterial Cellulose/Ag_2Se Nanocomposites. *ACS Appl Energy Mater* **5**, 3489-3501 (2022).

22. Xie J*, et al.* Flexible pCu_2Se-nAg_2Se thermoelectric devices *via in situ* conversion from printed Cu patterns. *Chem Eng J* **435**, 135172 (2022).

23. Yang D*, et al.* Flexible power generators by Ag_2Se thin films with record-high thermoelectric performance. *Nat Commun* **15**, 923 (2024).

24. Gao Q*, et al.* High Power Factor Ag/Ag_2Se Composite Films for Flexible Thermoelectric Generators. *ACS Appl Mater Interfaces* **13**, 14327-14333 (2021).

25. Liu Y*, et al.* Nanoengineering Approach toward High Power Factor Ag_2Se/Se Composite Films for Flexible Thermoelectric Generators. *ACS Appl Mater Interfaces* **15**, 36587-36593 (2023).

26. Li Y*, et al.* Exceptionally High Power Factor Ag_2Se/Se/Polypyrrole Composite Films for Flexible Thermoelectric Generators. *Adv Funct Mater* **32**, 2106902 (2022).

27. Du J*, et al.* Inkjet Printing Flexible Thermoelectric Devices Using Metal Chalcogenide Nanowires. *Adv Funct Mater* **33**, 2213564 (2023).

28. Kashyap A, Rawat D, Sarkar D, Singh NK, Biswas K, Soni A. Chemically Transformed Ag_2Te Nanowires on Polyvinylidene Fluoride Membrane For Flexible Thermoelectric Applications. *Angew Chem Int Ed* **63**, e202401234 (2024).

29. Zhang L*, et al.* N-type flexible Bi_2Se_3 nanosheets/SWCNTs composite films with improved thermoelectric performance for low-grade waste-heat harvesting. *Nano Energy* **104**, 107907 (2022).

30. Chang P-S, Liao C-N. Screen-printed flexible thermoelectric generator with directional heat

collection design. *J Alloys Compd* **836**, 155471 (2020).

31. Yusuf A*, et al.* Experimental and Theoretical Investigation of the Effect of Filler Material on the Performance of Flexible and Rigid Thermoelectric Generators. *ACS Appl Mater Interfaces* **13**, 61275-61285 (2021).

32. You H*, et al.* Flexible Bi_2Te_3-based thermoelectric generator with an ultra-high power density. *Appl Therm Eng* **202**, 117818 (2022).

33. Huo W, Xia Z, Gao Y, Guo R, Huang X. Flexible Thermoelectric Devices with Flexible Heatsinks of Phase-Change Materials and Stretchable Interconnectors of Semi-Liquid Metals. *ACS Appl Mater Interfaces* **15**, 29330-29340 (2023).

34. Zhu S*, et al.* Simultaneous Realization of Flexibility and Ultrahigh Normalized Power Density in a Heatsink-Free Thermoelectric Generator *via* Fine Thermal Regulation. *ACS Appl Mater Interfaces* **14**, 1045-1055 (2022).

35. Shi J*, et al.* Anisotropy engineering in solution-derived nanostructured Bi_2Te_3 thin films for high-performance flexible thermoelectric devices. *Chem Eng J* **458**, 141450 (2023).

36. Moiroux G, Tur C, Bourgault D, Garden JL. High temperature difference in a new flexible thermoelectric bismuth telluride microgenerator. *Sensor Actuat A-phys* **347**, 113961 (2022).

37. Mallick MM*, et al.* High Figure-of-Merit Telluride-Based Flexible Thermoelectric Films through Interfacial Modification *via* Millisecond Photonic-Curing for Fully Printed Thermoelectric Generators. *Adv Sci* **9**, 2202411 (2022).

38. Xu Q*, et al.* High-performance, flexible thermoelectric generator based on bulk materials. *Cell Rep Phys Sci* **3**, 100780 (2022).

39. Hou Y*, et al.* Whole Fabric-Assisted Thermoelectric Devices for Wearable Electronics. *Adv Sci*

9, 2103574 (2022).

40. Peng J*, et al.* 3D extruded composite thermoelectric threads for flexible energy harvesting. *Nat Commun* **10**, 5590 (2019).

41. Lee B*, et al.* High-performance compliant thermoelectric generators with magnetically self-assembled soft heat conductors for self-powered wearable electronics. *Nat Commun* **11**, 5948 (2020).

42. Jung S-J*, et al.* Porous organic filler for high efficiency of flexible thermoelectric generator. *Nano Energy* **81**, 105604 (2021).

43. Abe H, Takashiri M, Hara S, Arai T, Sasaki N, Tanaka S. Performance evaluation of flexible thermoelectric generator with Bi_2Te_3 thin-film. *Appl Therm Eng* **248**, 123258 (2024).

44. Park JW, Kim CS, Choi H, Kim YJ, Lee GS, Cho BJ. A Flexible Micro-Thermoelectric Generator Sticker with Trapezoidal-Shaped Legs for Large Temperature Gradient and High-Power Density. *Adv Mater Technol* **5**, 2000486 (2020).

45. Shang H*, et al.* $Bi_{0.5}Sb_{1.5}Te_3$-based films for flexible thermoelectric devices. *J Mater Chem A* **8**, 4552-4561 (2020).

46. Lincoln B, Annie Sujatha R, Veluswamy P, Majumdar A. A hybrid ceramic-based flexible thermoelectric nanogenerator with enhanced thermopower for human energy harvesting. *Energ Convers Manage* **292**, 117364 (2023).

47. Yang Y*, et al.* Stretchable Nanolayered Thermoelectric Energy Harvester on Complex and Dynamic Surfaces. *Nano Lett* **20**, 4445-4453 (2020).

48. Ao D-W*, et al.* Assembly-Free Fabrication of High-Performance Flexible Inorganic Thin-Film Thermoelectric Device Prepared by a Thermal Diffusion. *Adv Energy Mater* **12**, 2202731

(2022).

49. Lin S*, et al.* Flexible thermoelectric generator with high Seebeck coefficients made from polymer composites and heat-sink fabrics. *Commun Mater* **3**, 44 (2022).

50. Shi T*, et al.* Modifying carbon fiber fabric for flexible thermoelectric energy conversion. *Appl Surf Sci* **610**, 155479 (2023).

51. Pires AL*, et al.* Printed Flexible μ-Thermoelectric Device Based on Hybrid Bi_2Te_3/PVA Composites. *ACS Appl Mater Interfaces* **11**, 8969-8981 (2019).

52. Li Y*, et al.* A flexible thermoelectric device based on a Bi_2Te_3-carbon nanotube hybrid. *J Mater Sci Technol* **58**, 80-85 (2020).

53. Varghese T*, et al.* Flexible Thermoelectric Devices of Ultrahigh Power Factor by Scalable Printing and Interface Engineering. *Adv Funct Mater* **30**, 1905796 (2020).

54. Banerjee P*, et al.* Effect of particle-size distribution and pressure-induced densification on the microstructure and properties of printable thermoelectric composites and high energy density flexible devices. *Nano Energy* **89**, 106482 (2021).

55. Choo S*, et al.* Cu_2Se-based thermoelectric cellular architectures for efficient and durable power generation. *Nat Commun* **12**, 3550 (2021).

56. Yang D*, et al.* High thermoelectric performance of aluminum-doped cuprous selenide thin films with exceptional flexibility for wearable applications. *Nano Energy* **117**, 108930 (2023).

57. Cao J*, et al.* Flexible elemental thermoelectrics with ultra-high power density. *Mater Today Energy* **25**, 100964 (2022).

58. Van Toan N, Kim Tuoi TT, Ono T. High-performance flexible thermoelectric generator for self-powered wireless BLE sensing systems. *J Power Sources* **536**, 231504 (2022).

59. Toan NV, Tuoi TTK, Sui H, Trung NH, Samat KF, Ono T. Ultra-flexible thermoelectric generator based on silicone rubber sheet and electrodeposited thermoelectric material for waste heat harvesting. *Energy Rep* **8**, 5026-5037 (2022).

60. Lu Y*, et al.* Ultrahigh performance PEDOT/Ag_2Se/CuAgSe composite film for wearable thermoelectric power generators. *Mater Today Phys* **14**, 100223 (2020).

61. Na Y, Kim S, Mallem SPR, Yi S, Kim KT, Park K-I. Energy harvesting from human body heat using highly flexible thermoelectric generator based on Bi_2Te_3 particles and polymer composite. *J Alloys Compd* **924**, 166575 (2022).

62. Jiang C*, et al.* Ultrahigh performance polyvinylpyrrolidone/Ag_2Se composite thermoelectric film for flexible energy harvesting. *Nano Energy* **80**, 105488 (2021).

63. Yin L*, et al.* CALPHAD accelerated design of advanced full-Zintl thermoelectric device. *Nat Commun* **15**, 1468 (2024).

64. Yan B*, et al.* Heat-sink-free solar-driven thermoelectric device for micro energy scavenging. *Nano Energy* **123**, 109382 (2024).

65. Zeng C*, et al.* Kirigami-inspired organic and inorganic film-based flexible thermoelectric devices with built-in heat sink. *Nano Energy* **121**, 109213 (2024).

66. Liu Y*, et al.* Design and experimental study of a compact thermoelectric device driven by solar heating and radiative cooling. *Next Energy* **4**, 100146 (2024).
